# Supplementary material for: A genome-wide assessment of the ancestral neural crest gene regulatory network
Source: Nat Commun. 2019 Oct 16;10:4689. doi: 10.1038/s41467-019-12687-4 (PMC6795873; doi:10.1038/s41467-019-12687-4)
Supplement: Supplementary file 1 — Information [file 41467_2019_12687_MOESM1_ESM.pdf]

# A genome-wide assessment of the ancestral neural crest gene regulatory network

Dorit Hockman, Vanessa Chong-Morrison, Stephen A. Green, Daria Gavriouchkina,
Ivan Candido-Ferreira, Irving T.C. Ling, Ruth M. Williams, Chris T. Amemiya,
Jeramiah J. Smith, Marianne E. Bronner and Tatjana Sauka-Spengler*

---

Supplemental Material

---

* Lead contact and corresponding author: tatjana.sauka-spengler@imm.ox.ac.uk

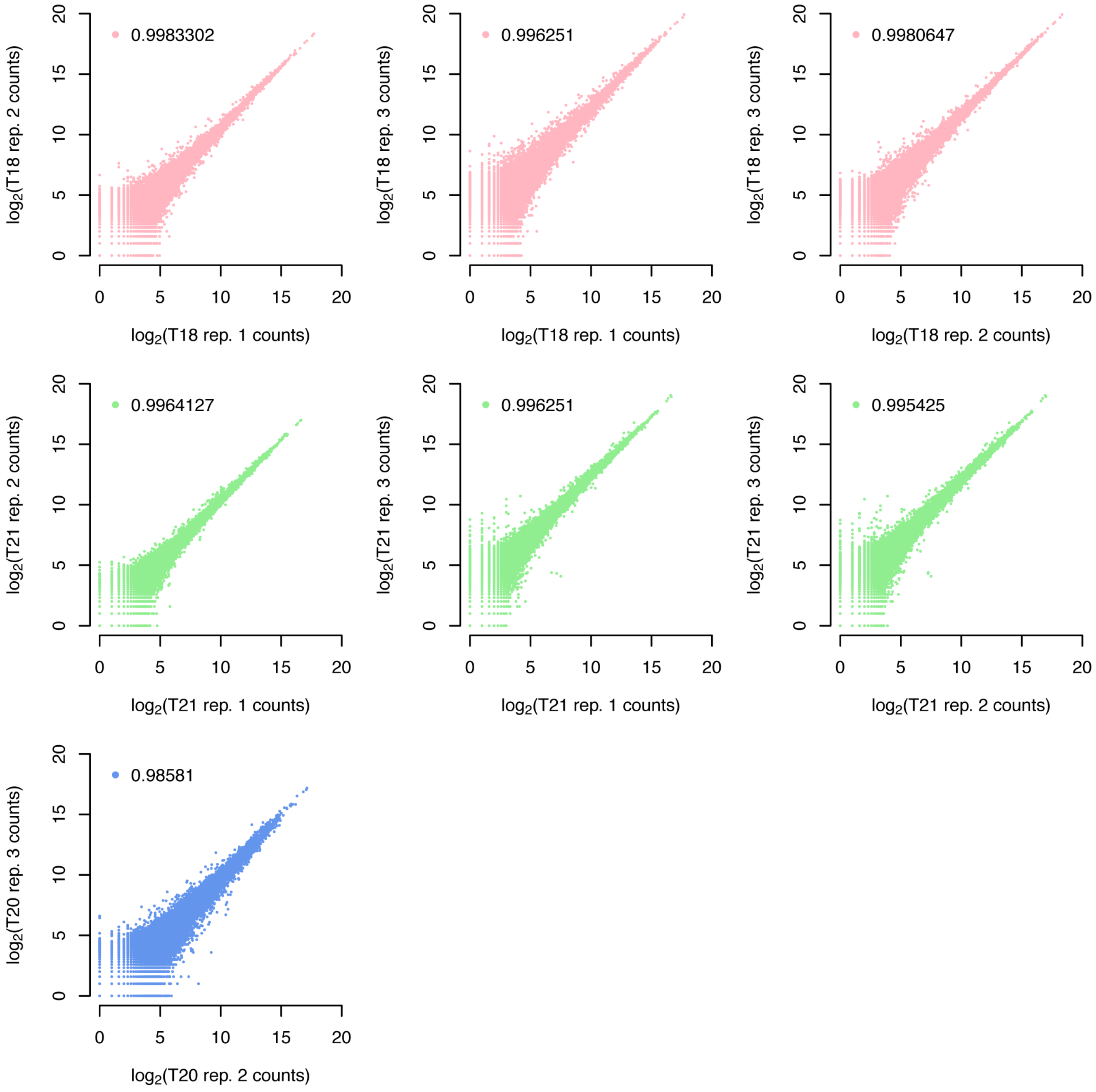


Supplementary Figure 1. **Reproducibility of RNA-seq experiments.** Scatterplots between replicate dorsal neural tube RNA-seq datasets at T18, T20 and T21 shows replicates are highly correlated. Pearson correlation coefficients (r) for all comparisons are given.

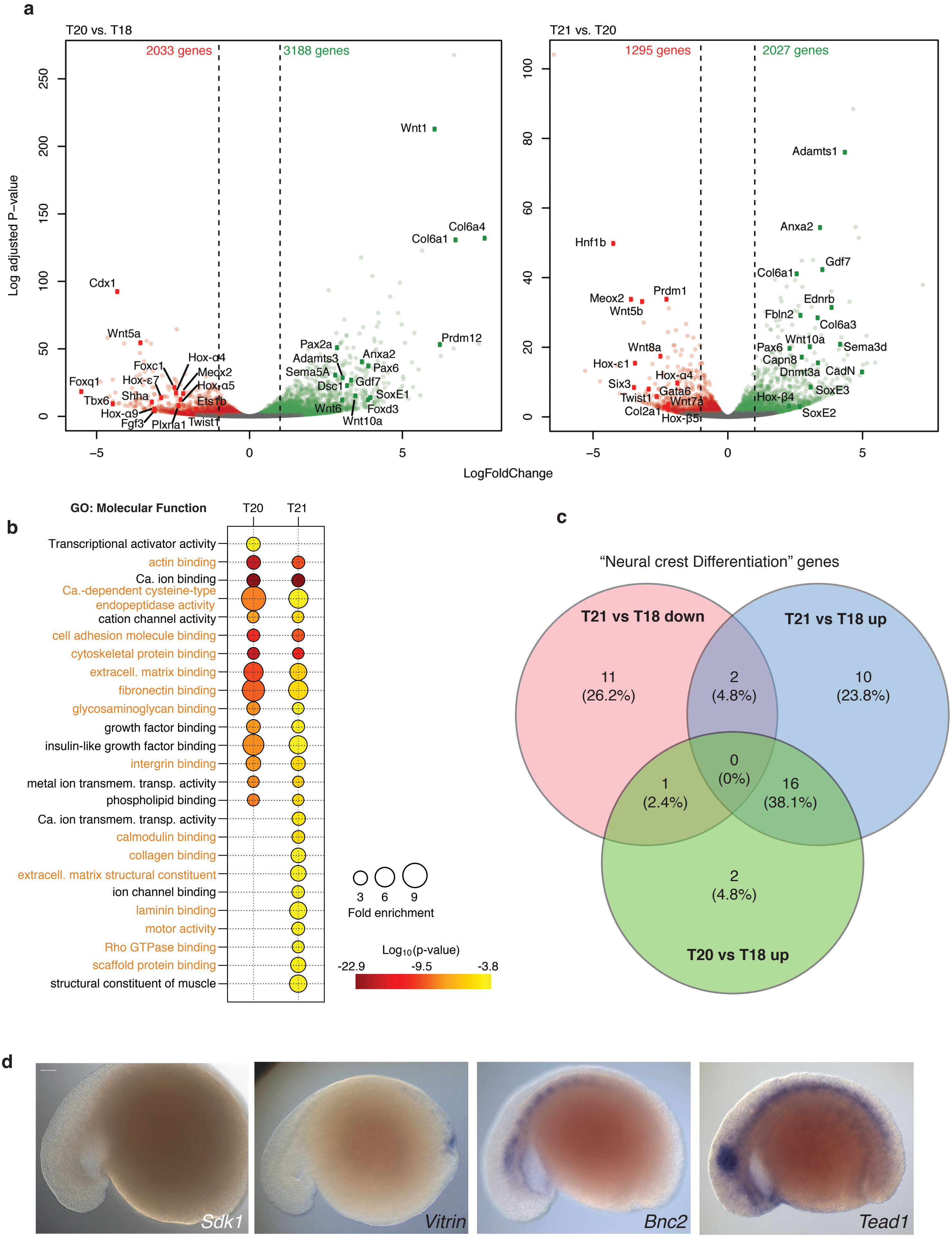


Supplementary Figure 2. **Dynamics of the developing neural crest gene expression profile cont.** a, Volcano plots of differential expression analysis between T20 and T18; and T21 and T20 (p-value < 0.05; green, enriched; red depleted at T21). Coloured dots and labels indicate genes previously known to be enriched or depleted in the developing neural crest. b, Bubble plots summarising enrichment and p-values for the most significant GO Molecular Function terms associated with enriched genes at T20 and T21 relative to T18 (only terms enriched more than three-fold are shown). Several GO terms associated with cell migration (orange) are present. c, Venn diagram showing the overlap of genes associated with the GO term "neural crest cell differentiation" in the indicated stage comparisons. d, Whole mount *in situ* hybridisation for the indicated genes at T21. Scale bar in d: 100 μm.

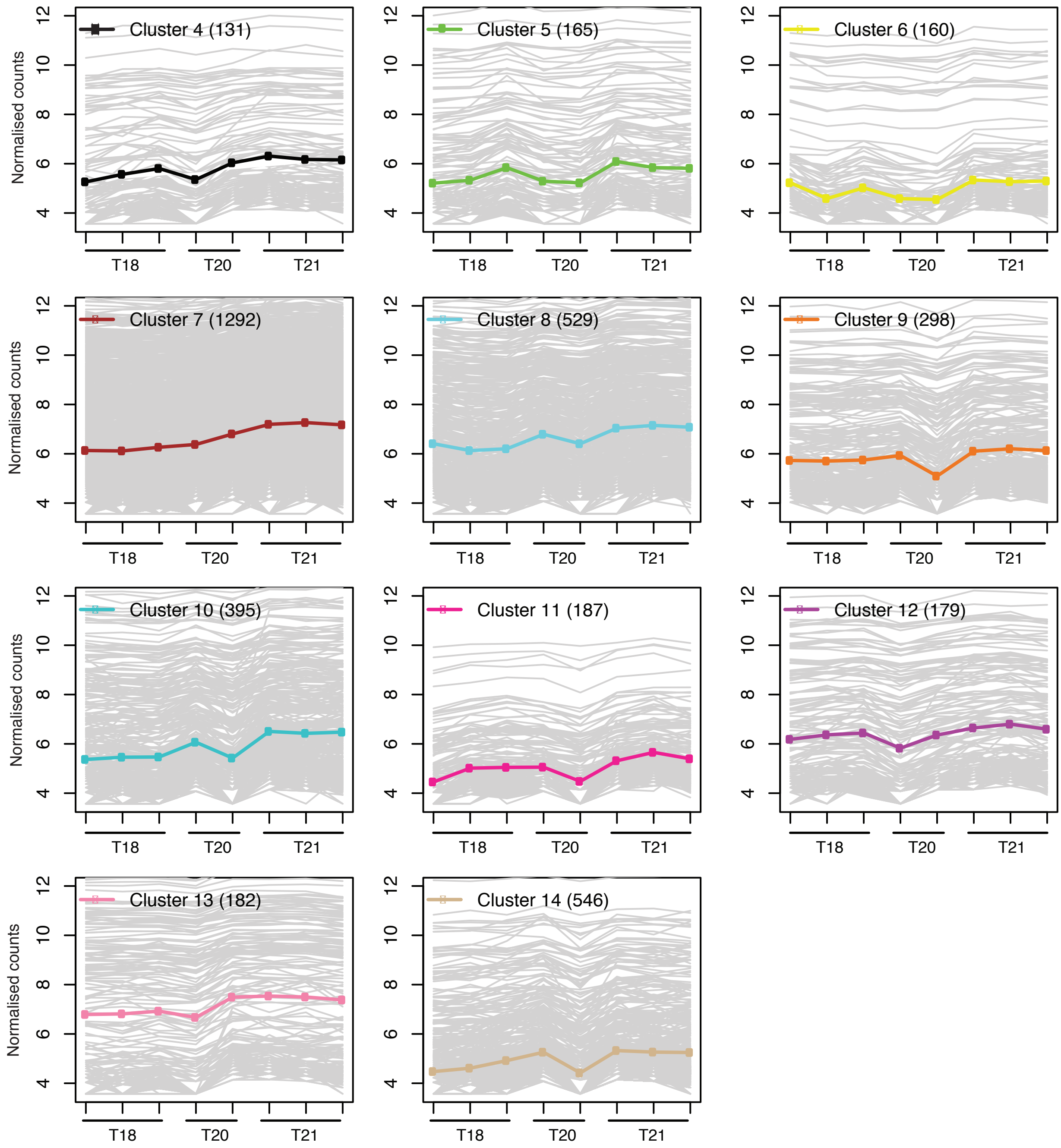


**Supplementary Figure 3. Additional WGCNA clusters.** Significantly higher gene expression in seen in these clusters at T21.

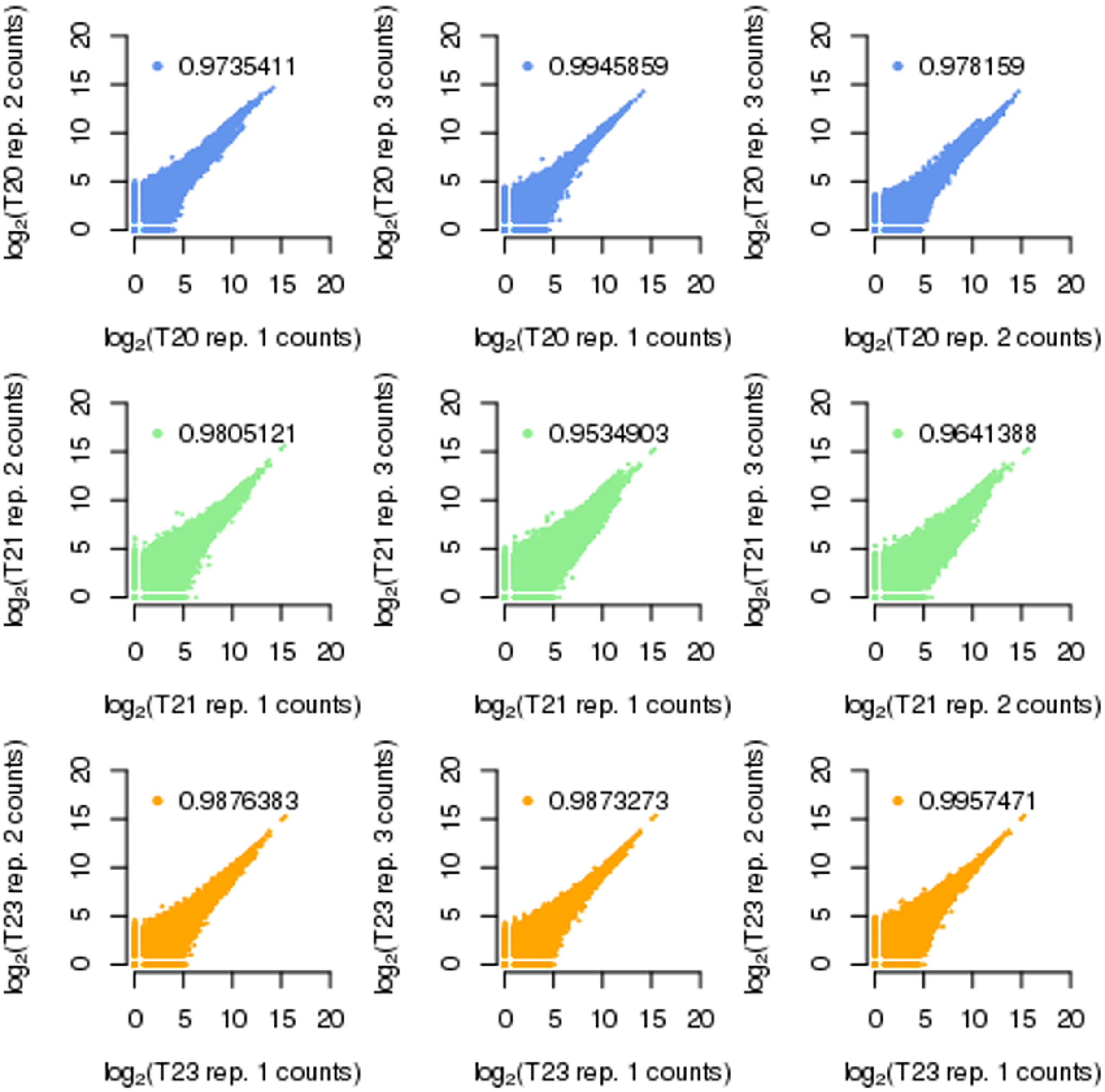


Supplementary Figure 4. **Reproducibility of ATAC-seq experiments.** Scatterplots between replicate dorsal neural tube (T20 and T21) and head (T23) ATAC-seq datasets shows replicates are highly correlated. Pearson correlation coefficients (r) for all comparisons are given.

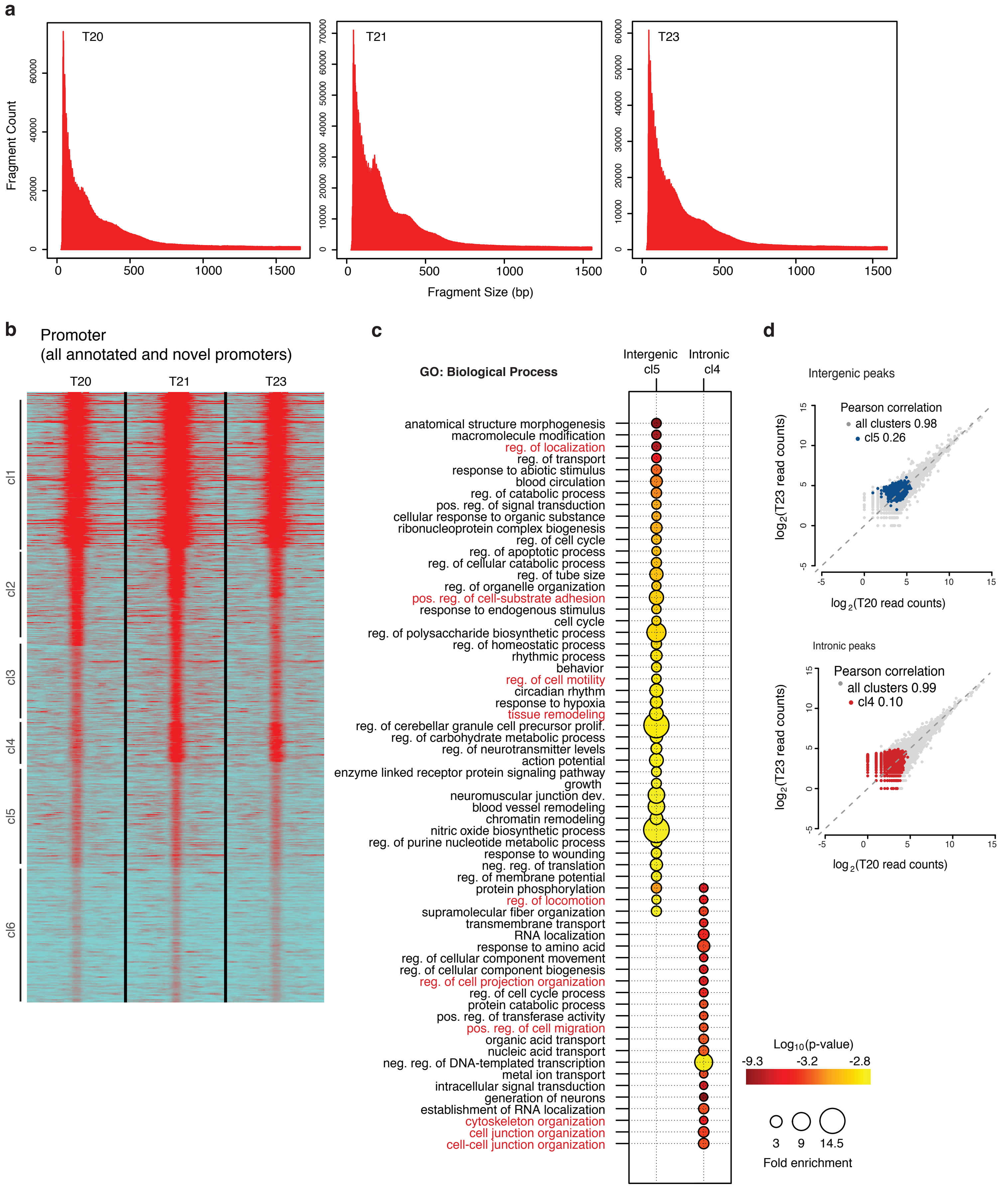


Supplementary Figure 5. **ATAC-seq analysis**. a, Histograms of fragments size for representative ATAC-seq samples at T20, T21 and T23 shows a periodicity of ~150 bp, corresponding to nucleosome protected fragments. b, Heatmap depicting k-means linear enrichment clustering of the promoter peakset (annotated and novel promoters) associated with the “Promoter” violin plot shown in Figure 2e. c, Bubble plots summarizing enrichment and p-values for the most significant GO biological process terms for the differentially expressed genes associated with Intergenic peaks k-means cluster 5 and Intronic peaks k-means cluster 4 (‘EMT’ clusters, see Fig. 2d). GO terms associated with cell migration are highlighted in red. Values shown are for terms that were more than 1.8-fold enriched. d, Scatterplot between T20 and T23 ATAC-seq read counts over consensus intergenic and intronic peaksets. Pearson correlation coefficients (r) for all clusters (grey) and for “EMT” clusters (intergenic cluster 5 in blue; intronic cluster 4 in red) are given.

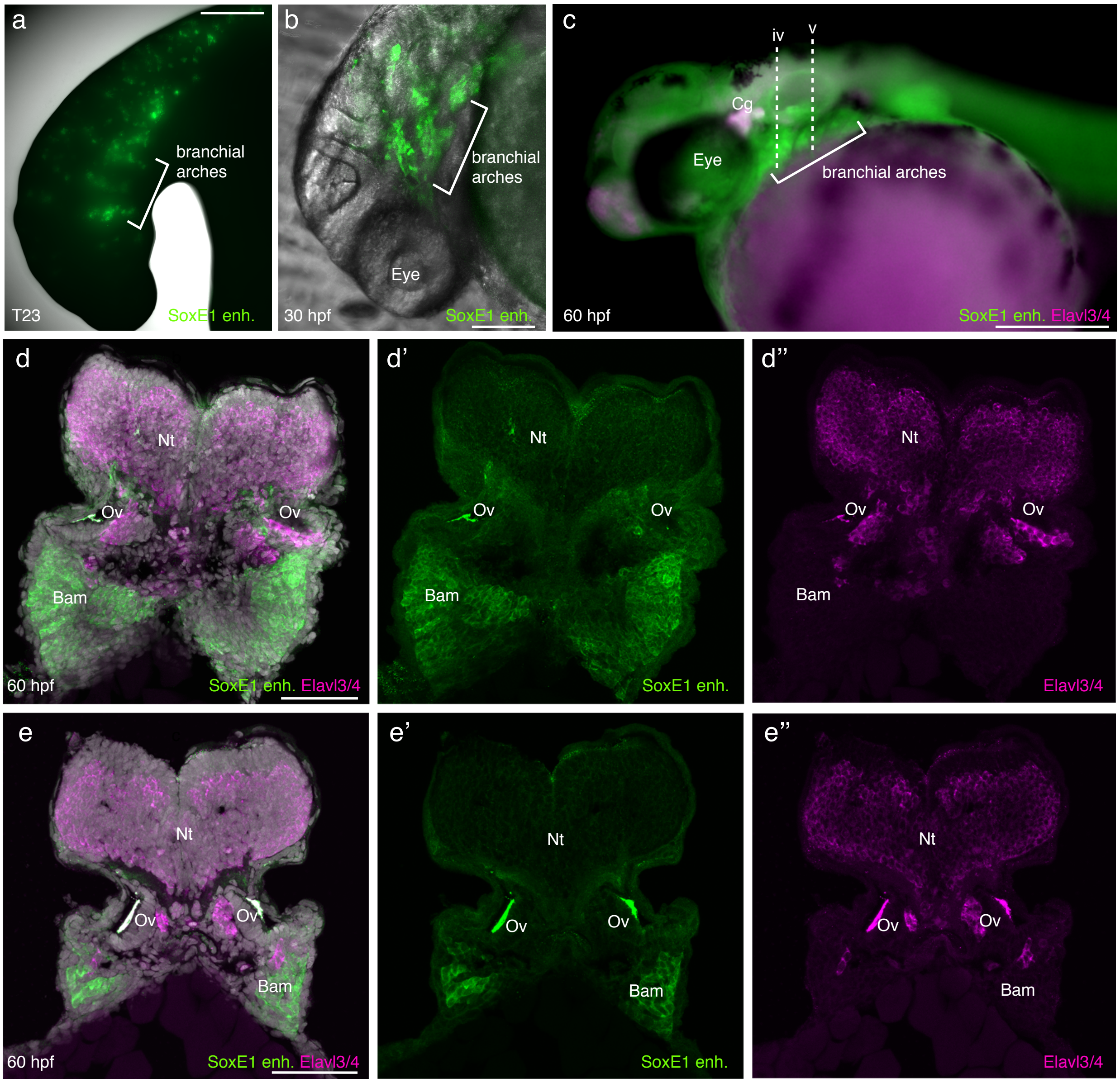


**Supplementary Figure 6. Sea lamprey SoxE1 enhancer activity is conserved in gnathostomes cont.** *SoxE1* enhancer-reporter expression in equivalently staged lamprey (T23, a) and zebrafish (30 hpf, b) embryos, as well as in a 60 hpf zebrafish embryo in wholemount (c) and section (d-e'') showing GFP expression in the developing branchial arches. At 60 hpf zebrafish, GFP expression is shown together with the post-mitotic neuronal marker, Elavl3/4 (magenta). Bam, branchial arch mesenchyme; Cg, cranial ganglia; Nt, neural tube; Ov, otic vesicle. Scale bars for wholemounts: 200 µm. Scale bars for sections: 50µm.

```
Pm_genome      1 GCGGTGGCGAGCCGAGTTCGACTTCAGCTCACGGCAAAAATCCCAACCAGTCCTGGAACT
Lp_ATAC        1 ................................................GTG.........
Lp_juv_cons    1 ------------------------------------------------------------

                                           SoxE
Pm_genome     61 CCCCCAAGGTCGACTTAACACCGGGGAGACAAAGGTGGCCCGGCATGTACGGTAATTGAA
Lp_ATAC       61 ...............C.....T............C.....K......C..A.........
Lp_juv_cons    1 ------------------------------....C............C..A.........

Pm_genome    121 CATCCACCTCATCCATAATAGAGGGTTTTTTGGGGTTAGATCGCGTAAATATATAAATGT
Lp_ATAC      121 .........MM.................................................
Lp_juv_cons   31 ......------------------------------------------------------

Pm_genome    181 GTCGTTAAAAACCCGCCACCACCCGACACAGCCAACTGGTAAGGGTTGTCACATAAACAG
Lp_ATAC      181 .....................................R......................
Lp_juv_cons   37 ------------------------------------------------------------

                                                                    Smad
Pm_genome    241 AACGTGCCAATTCGTTACTAGTACAGCACACCGGTGTTAAATCCACCTCATCGTCTGCCG
Lp_ATAC      241 ..............................Y...NNNNNNNNNNNNNNN...........
Lp_juv_cons   37 -----------------------------------------...................

Pm_genome    301 TTCTGGCGTTCGGAGATGCTCGCTACCAGCACTTGCCCCAACAGTCTGTTAAAGGCAACA
Lp_ATAC      301 .G.C...........G.................................C..........
Lp_juv_cons   56 .G.C...........G............................................

                                  SoxE
Pm_genome    361 TGGGGGTC--GCGGGGCTTTGTCTTCTGTACATCTGTATGGATGGCCACGGCAAGCGATT
Lp_ATAC      361 ......GG--.T.......................A.............K....NNNNNN
Lp_juv_cons  116 ......GGGG.T.......................A.............T....TT....

Pm_genome    419 GTGCCCAGCAGTCTCGGAGGAAGGGAGGTGGGGGGGGTGGGAATATCCGAATATTTATTT
Lp_ATAC      419 NNC....................A....N....T............G...T.........
Lp_juv_cons  176 ACC..A-...............-.AGA.G....T............G...T.........

Pm_genome    479 ATTTGTTGCCGGCTGGGGTCGCGGGGTTGGCTGGGAGGGGTTAACCATCTGCGGCATAAT
Lp_ATAC      479 ...................................T.......................A
Lp_juv_cons  234 ...................................T.......................A

Pm_genome    539 ACCGTGACTACAGCTGATGGGCGCGCGCGTGCACTCACAAGAGTCCAGTGTTGTAACTCC
Lp_ATAC      539 ..............................A..........C..................
Lp_juv_cons  294 ..............................A..........C..................

Pm_genome    599 CCGGGCCGCCTCCTCCCACGCTCGTGCTGCACCGCGGAAGCGTTCATCTCTTACAGGGCC
Lp_ATAC      599 .........................A..................................
Lp_juv_cons  354 .........................A..................Y...............

Pm_genome    659 TTGTTCATAATGCGGGCACTCCATCTCCCTTTTTGGCTCGCGCCGCTCTCCTCCCTCTCC
Lp_ATAC      659 ......C..........................K..........................
Lp_juv_cons  414 ......C..........................G.-.......................-

                                              Tfap2
Pm_genome    719 TCCCTCTCCTCCCTCTGCCATGGGAACTCCCCGAGGCTCTGGTCAGCGCGTTATTAACAG
Lp_ATAC      719 G...........................................................
Lp_juv_cons  472 --.GC.CT....................................................

Pm_genome    779 GAGGCAGGATGTGAGAGGTCAGGGCGGGGTGGGGGTAAGGGCAGCGAGGGGTGGATAGGG
Lp_ATAC      779 .............................G..............................
Lp_juv_cons  530 .............................G-.............................

Pm_genome    839 ACCCGTCTGTTCCTGGGGAGCAAGAGGCACCAGTGGGTGCATACCTCCACTCGCACGTGC
Lp_ATAC      839 ..GT.C.....................................................N
Lp_juv_cons  589 ..GT.C......................................................

Pm_genome    899 GCGCGCACTCGCGTGTGTGCGAGCGCGCACGCGAGTGTCCTGTGTTTGTGTGTGTGTGGG
Lp_ATAC      899 NNNNNNNNNNNNNNNNNNNNNNNNNNNNNNNNNNNNNNNNNNNNNNNNNNNNN.......
Lp_juv_cons  649 .A--------------------------------....GTCC...G.T............

Pm_genome    959 GAGCCGTGAAAGCACTTGCCCCAACAGTTTGTTCAGGCTATACGGGGGATATTTGTCTTT
Lp_ATAC      959 ....A..........................Y.S..........................
Lp_juv_cons  677 ....A..........................C.G..........................

Pm_genome   1019 GGAGCGGTAGGTGTAGTGGTTGGCGGCGCTGCGCTAACAACGTCGGTCTGCCGAGTTCGA
Lp_ATAC     1019 ..........T..............C...........G.....NNNNNNNNNNNNNNNNN
Lp_juv_cons  737 ..........T..............C-..........G..........G...........

Pm_genome   1079 TCCCCGCTCTCGGCCAACACAGCTGACGATTATCTGAAGCTGTCCTGGGCCTCCCCCTAG
Lp_ATAC     1079 NNNNNNNNNNNNNNNNN...C...............R....R..................
Lp_juv_cons  796 ..T.........A----------------.....C......A.....-.G.CT.......

Pm_genome   1139 GTTGACCCAACCTTAAATGAGTACCGTACTTGGCGGCGGTGTGTAAACATCGCCGCTCGA
Lp_ATAC     1139 ......T......................C...T....A.....................
Lp_juv_cons  839 ......T......................C...T....A.....................

                   SoxE
Pm_genome   1199 GAGACAAAGGCGGCCCGGCGTGATGCTGGCCATTCACCCCCCTCGTGTGCCGTACCCGGC
Lp_ATAC     1199 ......................G.........C........N...........G......
Lp_juv_cons  899 ......................G.........C.....-..............G......

                                                                          Hox
Pm_genome   1259 GTTCATAGGTGCTCGCTTACAGCACGTGCTCCTGCAGTCTTGTTAAGGCTACACGGGTGA
Lp_ATAC     1259 .................A.......T...C....T.........................
Lp_juv_cons  958 .................A.......T...C....T.........................

                   Hox
Pm_genome   1319 TCTATGGCCACTCTAGTTTGGCAATTGACATCTCTGTGTTTGTGTTGGTATCGTTAGACC
Lp_ATAC     1319 ......C......A..............T.........A....A.....C.....NNNNN
Lp_juv_cons 1018 ......C......A..............T.........A....A.....C..........

                                                               SoxE
Pm_genome   1379 GAGCCTTAAATGAGTAATATTATTGCGCTGCTTAAACGATGATCATTGTCATTGG
Lp_ATAC     1379 NNNNNNNNNNNNNNNNNNNNNNNN...T....................A......
Lp_juv_cons 1078 C..........A......CG.......T.........R..........A......
```

Supplementary Figure 7. **Multiple sequence alignment** showing the sea lamprey genome sequence (Pm_genome) in the region of the *SoxE1* enhancer aligned with the the brook lamprey ATAC-seq consensus sequence (Lp_ATAC) and the consensus sequence for three juvenile brook lamprey individuals (Lp_juv_cons). The region shown encompasses the ATAC-seq peaks in this region for both lampreyspecies. Putative neural crest transcription factor binding motifs identified as enriched above a random set of non-coding genomic regions are indicated by coloured boxes. Dots indicate conserved sequence. N's in the Lp_ATAC sequence indicate regions not covered by ATAC-seq data. Dashes in Lp_juv_cons indicate sequence gaps.

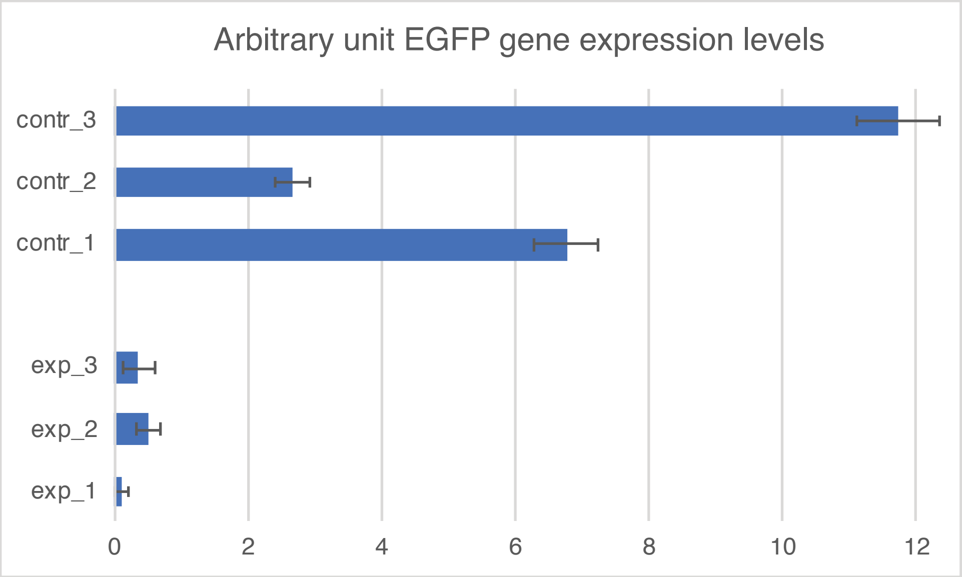


Supplementary Figure 8. **qPCR results for individual biological replicates** indicating the change in EGFP expression levels relative to bactin in the branchial arches after injection with either Cas9 mRNA alone (contr) or Cas9 mRNA together with sgRNAs against *hoxb2a*, *hoxb3a*, *hoxa2b*, *sox10* and *tfap2a* (exp). n=3 technical replicates per biological replicate; error bars: SD.

**Supplementary Tables**

**Supplmentary Table 1:** sgRNA design for CRISPR/Cas9 experiments in transgenic zebrafish

| Gene | Ensembl ID | cDNA length (bp) | Protein size (aa) | DNA-binding domain (aa) | DNA-binding domain (pos) | sgRNA spacer (BLAT good) | Target |
|---|---|---|---|---|---|---|---|
| hoxb2a | ENSDART00000146636 | 1832 | 409 | 175 - 239 [Homeobox domain] | Exon 2 of 2 | TACGTTTTTGCAGAGATTCA | SA/Exon 2 boundary |
| | | | | | | GTTTTTGCAGAGATTCAAGG | SA/Exon 2 boundary |
| hoxb3a | ENSDART00000078466 | 3411 | 417 | 178 - 242 [Homeobox domain] | Exon 5 of 5 | TTATTTCATTCCCACCCCAG | SA/Exon 5 boundary |
| | | | | | | CACCACCGCTGCTCTCCGCT | SA/Exon 5 boundary |
| hoxa2b | ENSDART00000009827 | 1914 | 363 | 131 - 195 [Homeobox domain] | Exon 2 of 2 | TAATGTTGTAATCCCTTCAT | SA/Exon 2 boundary |
| | | | | | | CTGCCACCATCAGAAATCTC | SA/Exon 2 boundary |
| sox10 | ENSDART00000112979 | 3205 | 485 | 88 - 180 [HMG box domain] | Exon 2 and 3 | CTGTGCTCCTCCGCCGACAT | ATG/Exon 2 boundary |
| | | | | | | CAGAACGAGTGGACCGATGT | ATG/Exon 2 boundary |
| tfap2a | ENSDART00000082349 | 2483 | 431 | Not known? 3 alt. start codons | Target exon 2 (shared) | TTATCGTTCTCTCTCTCCAA | SA/Exon 2 boundary |
| | | | | | | TGCCATTGCTGGTGCCGTCA | SA/Exon 2 boundary |

**Oligos for sgRNA template annealing, where (xxx…) denotes sgRNA spacer**

| | |
|---|---|
| Fw_oligo | GAAATTAATACGACTCACTATAGGG xxxxxxxxxxxxxxxxxxx GTTTTA GAGCTAGAAATAGC |
| Rv_oligo | AAAAGCACCGACTCGGTGCCACTTTTTCAAGTTG ATAACGGACTAGCCTTATTTTAACTTGCTATTTCTAGCTCTAAAAC |
