# Supplementary material for: A genome-wide assessment of the ancestral neural crest gene regulatory network
Source: Nat Commun. 2019 Oct 16;10:4689. doi: 10.1038/s41467-019-12687-4 (PMC6795873; doi:10.1038/s41467-019-12687-4)
Supplement: Supplementary file 6 — Reporting Summary [file 41467_2019_12687_MOESM6_ESM.pdf]

nature research

Corresponding author(s): Tatjana Sauka-Spengler

Last updated by author(s): Sep 19, 2019

Reporting Summary

Nature Research wishes to improve the reproducibility of the work that we publish. This form provides structure for consistency and transparency in reporting. For further information on Nature Research policies, see Authors & Referees and the Editorial Policy Checklist .

Statistics

For all statistical analyses, confirm that the following items are present in the figure legend, table legend, main text, or Methods section.

n/a	Confirmed	
☐	☒	The exact sample size (n) for each experimental group/condition, given as a discrete number and unit of measurement
☒	☐	A statement on whether measurements were taken from distinct samples or whether the same sample was measured repeatedly
☐	☒	The statistical test(s) used AND whether they are one- or two-sided<br>*Only common tests should be described solely by name; describe more complex techniques in the Methods section.*
☒	☐	A description of all covariates tested
☐	☒	A description of any assumptions or corrections, such as tests of normality and adjustment for multiple comparisons
☐	☒	A full description of the statistical parameters including central tendency (e.g. means) or other basic estimates (e.g. regression coefficient) AND variation (e.g. standard deviation) or associated estimates of uncertainty (e.g. confidence intervals)
☐	☒	For null hypothesis testing, the test statistic (e.g. F, t, r) with confidence intervals, effect sizes, degrees of freedom and P value noted<br>*Give P values as exact values whenever suitable.*
☒	☐	For Bayesian analysis, information on the choice of priors and Markov chain Monte Carlo settings
☐	☒	For hierarchical and complex designs, identification of the appropriate level for tests and full reporting of outcomes
☐	☒	Estimates of effect sizes (e.g. Cohen's d, Pearson's r), indicating how they were calculated

Our web collection on statistics for biologists contains articles on many of the points above.

Software and code

Policy information about availability of computer code

Data collection	No Software was used for data collection
Data analysis	The following software packages were used for data analysis and display in this study: FastQC, Sickle, STAR (v2.4.2), Subread featureCounts (v1.4.6-p4), Cufflinks Suite, DESeq2 (v.1.8.2), WGCNA, ggPlot, pheatmap, PANTHER Overrepresentation Test (V11), bedtools (v.2.15.0), blastx, Bowtie2, Picard (v1.83), BamTools, SAMtools, MACS2, HOMER (v4.7), SeqMINER

For manuscripts utilizing custom algorithms or software that are central to the research but not yet described in published literature, software must be made available to editors/reviewers. We strongly encourage code deposition in a community repository (e.g. GitHub). See the Nature Research guidelines for submitting code & software for further information.

Data

Policy information about availability of data

All manuscripts must include a data availability statement. This statement should provide the following information, where applicable:

- Accession codes, unique identifiers, or web links for publicly available datasets
- A list of figures that have associated raw data
- A description of any restrictions on data availability

The datasets generated during and/or analysed during the current study are available in the NCBI GEOarchive repository (accession number GSE112072).

Field-specific reporting

Please select the one below that is the best fit for your research. If you are not sure, read the appropriate sections before making your selection.

Life sciences study design

All studies must disclose on these points even when the disclosure is negative.

Sample size	Most analyses were done on a sample n=3-4, in one exceptional case we used a minimum sample size of n=2. This was due to sample availability.
Data exclusions	Variance stabilised normalised gene count tables generated by DESeq2 were filtered to only contain genes with a normalised count > 8 for subsequent WGCNA analysis to limit the number of genes analyses to < 46300 entries (the hightest number WGCNA could analyse at once).<br><br>Gene Ontology (GO) analysis was performed on annotated differentially expressed gene sets using the genes with baseMean value >20, log2FoldChange >1 for upregulated, and <-1 for downregulated genes and p-adjusted value <0.01 to limit analyses to significantly differentially expressed genes.<br><br>To generate ATAC-seq clusters by k-means clustering, intergenic and intronic peak-sets were further filtered to only contain peaks that were <50 000 bp away from genes that were enriched at stage T21 in comparison to T18. Promoter peaks were filtered to only include elements that overlapped a region of up to 2 kb upstream of the sea lamprey germline genome gene models.
Replication	Observations were only reported if they were observed in at least two samples.
Randomization	Samples were not allocated to experimental groups.
Blinding	Blinding was not relevant to this study as samples were not allocated to experimental groups.

Reporting for specific materials, systems and methods

We require information from authors about some types of materials, experimental systems and methods used in many studies. Here, indicate whether each material, system or method listed is relevant to your study. If you are not sure if a list item applies to your research, read the appropriate section before selecting a response.

Materials & experimental systems

n/a	Involved in the study	
☐	☒	Antibodies
☒	☐	Eukaryotic cell lines
☒	☐	Palaeontology
☐	☒	Animals and other organisms
☒	☐	Human research participants
☒	☐	Clinical data

Methods

n/a	Involved in the study	
☒	☐	ChIP-seq
☒	☐	Flow cytometry
☒	☐	MRI-based neuroimaging

Antibodies

Antibodies used	Alexa 488 conjugated anti-GFP antibody (Rabbit, Life Technologies; A21311); anti-GFP, (Rabbit, Torrey Pines, TP401); anti-Elavl3/4 (HuC/D) (mouse (IgG2b), Invitrogen, A-21271)
Validation	The GFP antibodies have been used in publications assessing GFP expression in transgenic zebrafish (https://www.thermofisher.com/antibody/product/GFP-Antibody-Polyclonal/A-21311 and https://www.acris-antibodies.com/antibodies/primary-antibodies/gfp-tp401.htm). The Invitrogen anti-Elavl3/4 antibody has been validated in zebrafish (https://www.thermofisher.com/antibody/product/HuC-HuD-Antibody-clone-16A11-Monoclonal/A-21271)

Animals and other organisms

Policy information about studies involving animals; ARRIVE guidelines recommended for reporting animal research

Laboratory animals	The following zebrafish (Danio rerio) lines (male and females) were used in this study: Tg(SoxE1_dwnstrm1-E1b:EGFP)ox 164 (generated for the purpose of this study) and Tg(tcf21:DsRed2)pd37.<br><br>Fertilised wild-type chicken eggs were obtained from Henry Stewart & Co (Norfolk).
Wild animals	Male and female adult sea lamprey (Petromyzon marinus) were supplied by the US Fish and Wildlife Service and Department of the Interior and housed in compliance with California Institute of Technology Institutional Animal Care and Use Committee protocol #1436. Adult sea lamprey die within weeks of releasing their gametes, thus all adult lamprey used in this study died while in captivity. Brook lamprey (Lampetra planeri) embryos and ammocoete larvae were collected from a shallow river in the New Forest National Park, United Kingdom, with permission from the Forestry Commission and maintained in filtered river water at 19C.

Field-collected samples	Lamprey were collected from the field as described above.
Ethics oversight	Work on sea lamprey was approved by the California Institute of Technology Institutional Animal Care and Use Committee protocol #1436.<br><br>Al brook lamprey embryo experiments were performed on embryos that had not yet reached free-feeding stages and thus were not regulated by the Animals (Scientific Procedures) Act 1986.<br><br>Zebrafish work was carried out in accordance to procedures authorized by the UK Home Office in accordance with UK law (Animals [Scientific Procedures] Act 1986) and the recommendations in the Guide for the Care and Use of Laboratory Animals.<br><br>All chick embryo experiments were performed on chicken embryos younger than 12 days of development and this were not regulated by the Animals (Scientific Procedures) Act 1986.

Note that full information on the approval of the study protocol must also be provided in the manuscript.
