# Supplementary material for: A genome-wide assessment of the ancestral neural crest gene regulatory network
Source: Nat Commun. 2019 Oct 16;10:4689. doi: 10.1038/s41467-019-12687-4 (PMC6795873; doi:10.1038/s41467-019-12687-4)
Supplement: Supplementary file 7 — Description of Additional Supplementary Files [file 41467_2019_12687_MOESM7_ESM.pdf]

Title: Supplementary data 1:
Description: Results of DESeq2 analyses comparing T18, T20 and T21 DNT RNA-seq datasets. XLOC numbers indicate de novo assembled transcripts. Gene annotations, when present, correspond to those given in the sea lamprey germline genome assembly. The presence of multiple gene annotations per transcript indicates that the assembled transcript overlaps with multiple individually annotated genes on the corresponding strand.

Title: Supplementary data 2:
Description: WGCNA clusters showing gene significance (GS; i.e. the correlation between the gene and the indicated trait [T18 or T21]), module membership (MM; i.e. the correlation of the module eigengene and the gene expression profile) and corresponding p-values (p.MM) for each gene in the cluster. XLOC numbers indicate de novo assembled transcripts. Annotations (GeneID), when present, correspond to those given in the sea lamprey germline genome assembly. The presence of multiple gene annotations per transcript indicates that the assembled transcript overlaps with multiple individually annotated genes on the corresponding strand. Cluster1 is shown in Fig. 1di, Cluster2 in Fig. 1dii and Cluster3 in Fig. 1diii. The remaining clusters are shown in Supplementary Fig. 3.

Title: Supplementary data 3:
Description: Genes associated with the GO term “NC differentiation” in the given stage comparisons.

Title: Supplementary data 4:
Description: Results of DESeq2 analyses for selected TFs used in silico two-way combinatorial TF analysis. These TFs displayed enriched motifs as well as enriched gene expression at T21 and were used to test for evidence of combinatorial TF activity at putative cis-regulatory elements.
